# Supplementary material for: Patterns of Intron Gain and Loss in Fungi
Source: PLoS Biol. 2004 Nov 30;2(12):e422. doi: 10.1371/journal.pbio.0020422 (PMC532390; doi:10.1371/journal.pbio.0020422)
Supplement: Table S1 — Also available at http://genes.mit.edu/NielsenEtAl/. (4.3 MB ZIP). [file pbio.0020422.st001.zip › NielsenEtAl/html/610.html]

AN7579.1.NCU02982.1.MG04506.1.FG01123.1


```
 CLUSTAL W (1.82) Multiple Sequence Alignments - Introns Inserted


Sequence 1: NCU02982.1	303 aa
Sequence 2: FG01123.1	288 aa
Sequence 3: MG04506.1	294 aa
Sequence 4: AN7579.1	238 aa
Alignment Length: 305 aa
Number Identitical Residues: 101 aa
Alignment Score (without introns) 5773


MG04506.1 	MPLEAVMIVVDNSESSRNGDYTPTRFDAQADAVNVLFQHVTNGNPESSVGLMSMGGKDPE
NCU02982.1	MPLEAVMIVVDNSESSRNGDYIPTRYDAQADAVNIIFQNVVQGNPESSVGLMSMAGRGPE
FG01123.1 	MVLEAVMVVVDNSESSRNGDYQPTRFDSQVDAVNITFQSITQGNPESSVGLMSMGGKGPE
AN7579.1  	----------------------------------------MRAHPQSAVGLMSMGGKGPE
          	                                         ..:*:*:******.*:.**

MG04506.1 	VLVTLTSDQGKILEGLHRTKKKVSGSSHLATAIQVAS0LALKHRQNTTQRTRIVAFVCSP
NCU02982.1	VLVTLTTDQGKILEGLHRTKKNIRGSAQLSRAIQVAW0LALKHRQNKSQRGRIVIFVCSP
FG01123.1 	VLVTLTTEQGKILEGLHRTKKKIGGSSHLKTGIQVAT0LALKHRQNRSQRQRIIVFVCSP
AN7579.1  	VLSTFTTDFGGILAGLHRTK--IHGTAHFTSSIQVAG0LALKHRSEKSQRQRIIVFSCSP
          	** *:*:: * ** ******  : *::::  .****  ******.: :** **: * ***

MG04506.1 	VSDEQKALVQLAGKLKKNNVTVDFVLFGDLDDDTSK-KLEAFNAKVKNNEGNSHLVVIPP
NCU02982.1	IEEEESDLVKLAKKMKKNNTSVDFVLFGDIDDENQK-KLEAFNREVKSGEG-SNMVVIPP
FG01123.1 	VEESEKELTTLAKKMKKANISVDFVLFGDLDDDSTKNKLQLFIDTVKTNEG-CHLVVIPP
AN7579.1  	IAEDEKTLVKLAKKMKKNNVSIDVIAFGDLESDQTK-KLEAFVENVKGGDG-SNLAIIPP
          	: :.:. *. ** *:** * ::*.: ***::.:  * **: *   ** .:* .::.:***

MG04506.1 	SGNLLSDQLIATPIF-GEG-AGSSAAAGGGGGG--GDFGDLEFDPATDPELALALRMSLE
NCU02982.1	SSKLLSDQLISTPILLGEAAAGGSGGAGGDAGGAANEDFPFGIDPSVDPELALALRMSME
FG01123.1 	SSKLLSDQLVSSPILLGEN-AGGSGGAGGAGGG--NDEFEFGFDPAMEPELALALRMSME
AN7579.1  	GPNLLSEELQASPILGGDG-SGGAGGLGGGDGGD-GGAFDLDAAAENDPELAFALRLSLE
          	. :***::* ::**: *:  :*.:.. **  **  .    :   .  :****:***:*:*

MG04506.1 	EENARKEKEAKKEAEQAKKQGLESVEEQD-ESAPLLDKSGGPSGSGSK---DK--KSDGG
NCU02982.1	EEKARQEKRAKEEAEAAKKASLEDVKEEAGESEPLLGKDGEPSGSGSKKDGDKPGKSDDG
FG01123.1 	EEKARQEKAAREEEEAAKKASLGDVKEED-----------EGQGSSSK-DQDK-DKKGDG
AN7579.1  	EEKNRQEKERREREAQQGKENLEGIPEEG---------------SSSK----K--DNEDP
          	**: *:**  ::.     * .* .: *:                *.**    *  .. . 

MG04506.1 	DKMDTS
NCU02982.1	DKMDTS
FG01123.1 	DKMDIS
AN7579.1  	DKMDTA
          	**** :
```
